# Cellular RNA Targets of Cold Shock Proteins CspC and CspE and Their Importance for Serum Resistance in Septicemic Escherichia coli

**DOI:** 10.1128/msystems.00086-22

**Published:** 2022-06-13

**Authors:** Yael Yair, Charlotte Michaux, Dvora Biran, Jörg Bernhard, Jörg Vogel, Lars Barquist, Eliora Z. Ron

**Affiliations:** a The Shmunis School of Biomedicine and Cancer Research, George S. Wise Faculty of Life Sciences, Tel-Aviv University, Tel Aviv, Israel; b RNA Biology Group, Institute of Molecular Infection Biology, University of Würzburg, Würzburg, Germany; c Institute for Microbiology, Ernst-Moritz-Arndt-Universität, Greifswald, Germany; d Helmholtz Institute for RNA-based Infection Research (HIRI), Helmholtz Center for Infection Research (HZI), Würzburg, Germany; e Faculty of Medicine, University of Würzburg, Würzburg, Germany; California State University, Fresno

**Keywords:** *Escherichia coli*, ExPEC, extraintestinal pathogenic *E. coli*, cold shock proteins, RNA binding proteins, CspC, CspE, serum resistance

## Abstract

The RNA chaperones, cold shock proteins CspC and CspE, are important in stress response and adaptation. We studied their role in the pathogenesis of a virulent Escherichia coli, representative of extraintestinal pathogenic E. coli (ExPEC) which are serum resistant and septicemic. We performed a global analysis to identify transcripts that interact with these cold shock proteins (CSPs), focusing on virulence-related genes. We used CLIP-seq, which combines UV cross-linking, immunoprecipitation and RNA sequencing. A large number of transcripts bound to the CSPs were identified, and many bind both CspC and CspE. Many transcripts were of genes involved in protein synthesis, transcription and energy metabolism. In addition, there were virulence-related genes, (i.e., *fur* and *ryhB*), essential for iron homeostasis. The CLIP-seq results were validated on two transcripts, *clpX* and *tdcA*, reported as virulence-associated. Deletion of either CspC or CspE significantly decreased their transcript levels and in a double deletion mutant *cspC/cspE*, the transcript stability of *tdcA* and *clpX* was reduced by 32-fold and 10-fold, respectively. We showed that these two genes are important for virulence, as deleting either of them resulted in loss of serum resistance, a requirement for sepsis. As several virulence-related transcripts interact with CspC or CspE, we determined the importance of these proteins for growth in serum and showed that deletion of either gene significantly reduced serum survival. This phenotype could be partially complemented by *cspE* and fully complemented by *cspC*. These results indicate that the two RNA chaperones are essential for virulence, and that CspC particularly critical.

**IMPORTANCE** Virulent Escherichia coli strains that cause infections outside the intestinal tract—extraintestinal pathogenic E. coli (ExPEC)—constitute a major clinical problem worldwide. They are involved in several distinct conditions, including urinary tract infections, newborn meningitis, and sepsis. Due to increasing antibiotic resistance, these strains are a main factor in hospital and community-acquired infections. Because many strains, which do not cross-react immunologically are involved, developing a simple vaccine is not possible. Therefore, it is essential to understand the pathogenesis of these bacteria to identify potential targets for developing drugs or vaccines. One of the least investigated systems involves RNA binding proteins, important for stability of transcripts and global gene regulation. Two such proteins are CspC and CspE (“cold shock proteins”), RNA chaperones involved in stress adaptation. Here we performed a global analysis to identify the transcripts which are affected by these two chaperones, with focus on virulence-associated transcripts.

## INTRODUCTION

In bacteria, RNA binding proteins (RBPs) are involved in posttranscriptional regulation, and thus are central in the response to environmental changes and stress. RBPs regulate various cellular mechanisms such as the translation, degradation, stabilization, and processing of RNA (1, 2). One group of important RBPs are cold shock proteins (CSPs), nine of which are encoded by Escherichia coli and termed CspA to CspI (3). CspA was the first cold shock protein to be discovered (4) and its cold-inducibility gave the family its name, although only CspA, CspB, CspE, CspG, and CspI are cold-inducible. Members of the CSP family act as RNA chaperones (3) that bind nucleic acids (DNA and RNA) with low-sequence specificity and low-binding affinity, and are involved in mRNA stability and translation (5). Two important CSPs are CspC and CspE, central proteins in cellular stress response and adaptation to environmental changes (6). Well-studied cases of posttranscriptional regulation by CspC and CspE in E. coli are the universal stress protein A (UspA) and RpoS, the general stress-response RNA polymerase sigma factor (7). CspC and CspE are highly similar in amino acid sequence, with only eight amino acid differences between them (6), and both are constitutively expressed at 37°C (8). There is a compensatory dynamic between CspC and CspE—deleting CspC results in an overproduction of CspE, and vice versa (6). The two CSPs were found to have transcription antitermination activity, with the strongest evidence for CspE (9, 10).

In contrast to their high sequence identity and many shared targets, CspC and CspE have different regulatory and biological functions. The cellular levels of CspC are not affected by cold shock, its levels are increased in stationary phase, and are reduced upon heat shock, due to proteolysis (6, 11) affecting the stability of the mRNA of the relevant stress genes such as *dnaK*, *groEL*, *hslVU*, and *htpG* (12). CspE is a negative regulator of CspA (13), known to be involved in RNA condensation and cell division (13), and is cold-inducible. Other than cold shock, its levels are relatively constant upon a change in environmental conditions (14). Its transcript levels are modulated through temperature-dependent regulatory structures in the *cspE* leader sequences to control translation efficiency and differential RNA stability (14). Taken together, it appears that CspC is a modulator of transcript stability upon exposure to environmental stress while CspE acts more as a “housekeeping RNA chaperone” with some involvement in cold shock response.

The first evidence of a possible role in virulence for CspC and CspE was shown in Salmonella enterica serovar Typhimurium (15). Extensive RNA ligand profiling performed for all six Salmonella CSPs with RIP-seq experiments indicated that CspC and CspE are involved in various stress responses and virulence. In Salmonella, CspC and CspE affect gene expression during host cell infection, are involved in motility and biofilm formation and are crucial to pathogenicity. Another recent study supporting the role of CspE in pathogenicity showed that CspE is essential for motility and biofilm formation in Salmonella, with mediation of this process by CspA (16).

Here, we have profiled transcript binding by CspC and CspE, in the septicemic extraintestinal pathogenic E. coli (ExPEC) O78:H19 ST88 isolate 789 (O78-9). This analysis was performed using CLIP-seq, which combines UV cross-linking with immunoprecipitation followed by RNA sequencing, to identify transcripts that are regulated by CspC and CspE, the “CspC/E regulon.” This is the first time the CLIP-seq method has been used to profile the interactions of cold shock proteins with cellular transcripts. The results provided evidence that several gene involved in virulence and essential for survival in serum, a prerequisite for septicemia, are also stabilized by CspC and CspE. These results indicate that these two RNA chaperones are essential for virulence. Indeed, a deletion of either *cspC* or *cspE* resulted in complete serum sensitivity, which could be fully complemented by *cspC*.

## RESULTS

### Global identification of RNA molecules bound by CspC and CspE.

**FIG 1:**
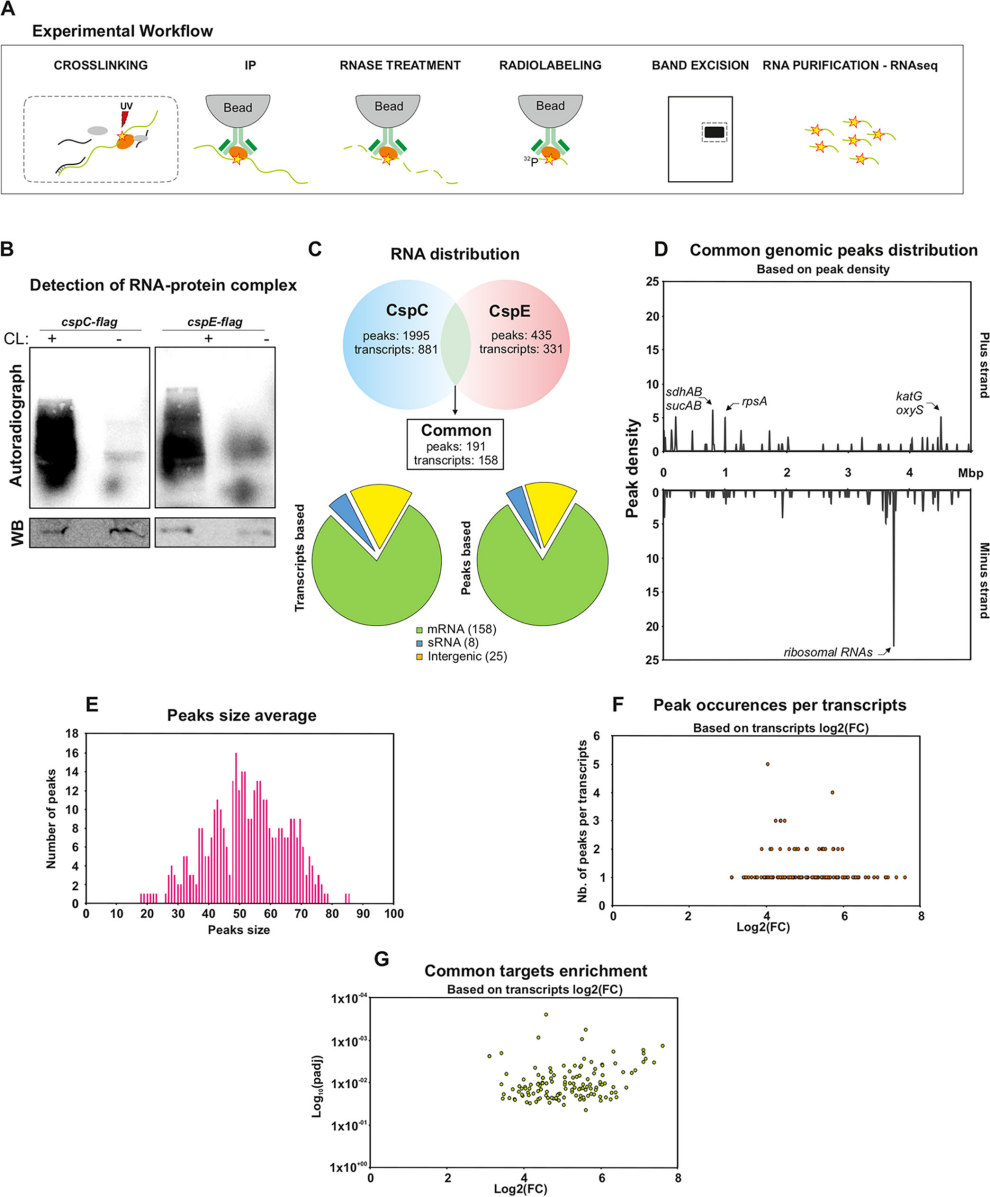
CLIP-seq of CspC and CspE in Escherichia coli O789. (A) Schematic representation of the experimental workflow. (B) Detection of cross-linked, immunoprecipitated and radioactively labeled CspC-RNA or CspE-RNA complexes. Radioactive signals were detected by phosphorimaging, whereas detection of the CspC-flag or CspE-flag proteins, used as control for the immunoprecipitation, were undertaken by Western blotting (WB). (C) RNA distribution of the common peaks and transcripts between CspC and CspE. A peak is considered common if its start and stop are the same for both proteins plus or minus 5 nt. (D) Genomic location of the common peaks along the E. coli genome with the peaks present on the positive strand on the top and the peaks present on the negative strand on the bottom. Mbp, millions of basepairs. (E) Size average of the common peaks. (F) Volcano plot representing the common transcripts, based on their peaks with on the *x* axis the transcripts log 2-fold change and on the *y* axis the p-adjusted value plotted on a logarithm scale. (G) Peaks occurrences per transcripts. The *x* axis represents the log 2-fold change of the transcripts while the *y* axis represents the number of peaks on each individual transcript.

**FIG 2:**
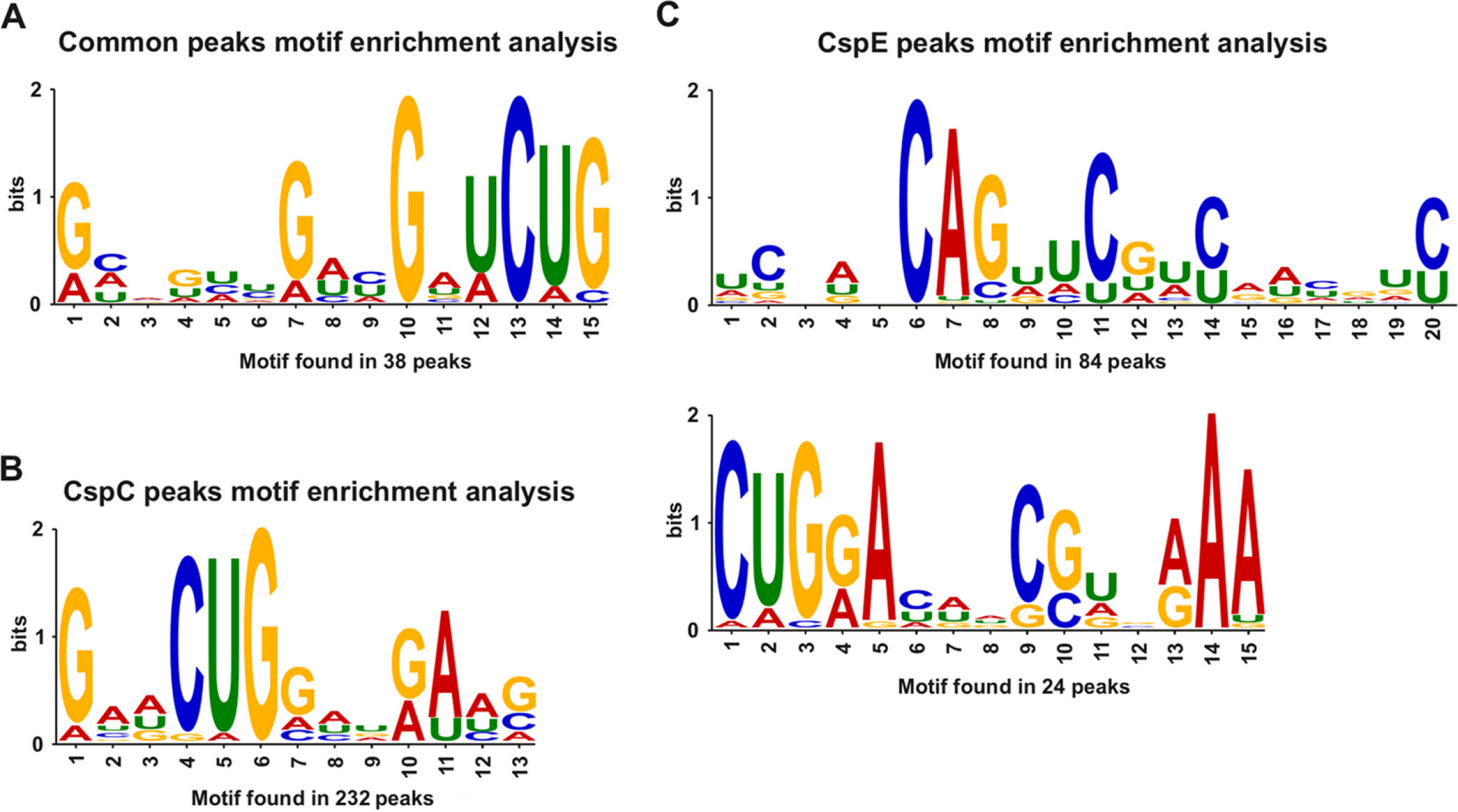
Motif found by MEME motif search analysis. The CSPs 15-bp consensus sequence was downloaded from Meme Suite. (A) RNA motif found in common peaks, with 38 peak sequences, were included in this analysis. (B) RNA motif found for CspC, with 232 peak sequences, were included in this analysis. (C) RNA motifs found for CspE. The upper motif was found in 84 peak sequences; bottom motif was found in 24 peak sequences.

The goal of this work was to investigate gene regulation by CspC and CspE, and to study their functions in a pathogenic strain of E. coli. For a global systematic approach, we co-immunoprecipitated the RNA-CSP complexes after stabilization by UV cross-linking, as illustrated in Fig. 1A and Fig. 2B. Briefly, the cells were lysed and immunoprecipitated using specific antibodies and the bound RNA was trimmed leaving only the region surrounding the direct interaction site, labeled, and analyzed (Fig. 1B) This method (CLIP-seq) brings new insights by mapping transcriptome-wide interactions of RBPs and their targets, and has been successfully applied to study the roles of central RBPs such as Hfq and CsrA (17) and ProQ (18). Here, we applied CLIP-seq to an ExPEC strain associated with sepsis, O78-9, grown in rich medium at 37°C to early stationary phase. Under these conditions both CSPs are highly expressed (6).

CLIP-seq analysis yielded a total of 2,430 peaks, of which 191 are shared between the two CSPs. For CspC, 1,995 peaks were detected, and they appear in 681 transcripts, 30 sRNAs, and 166 intergenic regions without a known annotation (padj < 0.05; Table S1; Fig. S1A). For CspE, 435 peaks were detected, and they appear in 214 transcripts, 11 sRNAs, and 64 intergenic regions without a known annotation (padj < 0.05; Table S2; Fig. S2A). The number of potential targets found for CspE was significantly lower, compared with CspC, and it is unclear whether this is due to a technical issue or if it reflects a biological property.

10.1128/msystems.00086-22.1 FIG S1 (A) RNA distribution of the common peaks and transcripts of CspC. (B) Size average of the peaks of CspC. (C) Genomic distribution of the CspC peaks with on the right the peak density (y axis) and on the left the fold change (y axis). Both x axis represent the genomic location and both graphs on the top represents the peaks of the positive strand and both graph on the bottom the ones of the negative strand. (D) Plots representing the CspC interactome enrichment. On the upper right panel, volcano plot of the peaks with on the x axis the transcripts log 2-fold-change and on the y axis the P-adjusted value plotted on a logarithm scale. On the upper left, similar volcano plot but this time based on the transcripts. On the bottom right, peaks occurrences per transcripts. The x axis represents the log 2-fold-change of the transcripts while the y axis represents the number of peaks on each individual transcript. On the bottom left, plot illustrating the correlation between the number of peaks per transcripts (y axis) and the actual length of each individual transcripts (x axis). (E) Gene ontology enrichment analysis performed by Panther where five main annotation sets are represented (biological process; molecular function; cellular component; protein class and pathway). The x axis represents the enrichment fold (*P*-value <0.05) of each pathway. The y axis represents the enriched pathways, classified by color based on their annotation set. Download FIG S1, PDF file, 2.2 MB. Copyright © 2022 Yair et al. 2022 Yair et al. https://creativecommons.org/licenses/by/4.0/ This content is distributed under the terms of the Creative Commons Attribution 4.0 International license.

10.1128/msystems.00086-22.2 FIG S2 **(**A) RNA distribution of the common peaks and transcripts of CspE. (B) Size average of the peaks of CspE. (C) Genomic distribution of the CspE peaks with on the right the peak density (y axis) and on the left the fold change (y axis). Both x axis represent the genomic location and both graphs on the top represents the peaks of the positive strand and both graph on the bottom the ones of the negative strand. (D) Plots representing the CspE interactome enrichment. On the upper right panel, volcano plot of the peaks with on the x axis the transcripts log 2-fold-change and on the y axis the P-Adjusted value plotted on a logarithm scale. On the upper left, similar volcano plot but this time based on the transcripts. On the bottom right, peaks occurrences per transcripts. The x axis represents the log 2-fold-change of the transcripts while the y axis represents the number of peaks on each individual transcript. On the bottom left, plot illustrating the correlation between the number of peaks per transcripts (y axis) and the actual length of each individual transcripts (x axis). (E) Gene ontology enrichment analysis performed by Panther where four main annotation sets are represented (biological process, molecular function, cellular component, protein class). The x axis represents the enrichment fold (*P*-value <0.05) of each pathway. The y axis represents the enriched pathways, classified by color based on their annotation set. Download FIG S2, PDF file, 1.5 MB. Copyright © 2022 Yair et al. 2022 Yair et al. https://creativecommons.org/licenses/by/4.0/ This content is distributed under the terms of the Creative Commons Attribution 4.0 International license.

There are 191 peaks shared by the two CSPs (Fig. 1C; Table S3). Further analysis of common peaks distribution across the O78-9 genome revealed a significant enrichment of peaks on the negative strand, which corresponds to the location of ribosomal genes, whereas on the positive strand, peaks are preferentially found in genes related to metabolism and respiration (*sdhAB* and *sucAB* operons), as well as genes involved in oxidative stress (*katG* and *oxyS*) (Fig. 1D) with an average peak size of 53 nt (Fig. 1E). It should be noted that these observations of peak size and enrichment locations are also true for peaks of CspC and CspE individually (Fig. S1B and C; Fig. S2B, C). Most common targets were found to be bound by the CSPs in one or two binding sites (Fig. 1F), with an enrichment of 4- to 6-log_2_-fold change (Fig. 1G). Few transcripts, mainly genes encoding ribosomal proteins, harbored more than two peaks (Fig. 1F) which could be explained by the abundance of those transcripts and the higher chance they have to get cross-linked at multiple sites. Details of the peaks occurrence per transcripts and their enrichments for CspC and CspE are also presented in Fig. S2D and 3D, respectively.

10.1128/msystems.00086-22.3 TABLE S1 CspC peaks from the CLIP analysis. The data of the three biological replicate for both cross-linking and non-cross-linking conditions are displayed with features such as the peak strand, fold change, p-value, location, and peak length. Download Table S1, CSV file, 1.7 MB. Copyright © 2022 Yair et al. 2022 Yair et al. https://creativecommons.org/licenses/by/4.0/ This content is distributed under the terms of the Creative Commons Attribution 4.0 International license.

10.1128/msystems.00086-22.4 TABLE S2 CspE peaks from the CLIP analysis. The data of the three biological replicate for both cross-linking and non-cross-linking conditions are displayed with features such as the peak strand, fold change, p-value, location, and peak length. Download Table S2, CSV file, 1.4 MB. Copyright © 2022 Yair et al. 2022 Yair et al. https://creativecommons.org/licenses/by/4.0/ This content is distributed under the terms of the Creative Commons Attribution 4.0 International license.

10.1128/msystems.00086-22.5 TABLE S3 Common peaks shared between CspC and CspE, based on their CspC sequences. A peak was considered common if its start and stop are the same for both proteins plus or minus 5 nt. The first table sheet includes the common peaks location, strand, start and end, their fold change and p-value. The other table sheets are the peaks details for CspC and CspE classified according to their genomic or plasmidic location, CP010315 for the chromosome, CP010316 for the plasmid 1 (pAPEC_078.1), CP010317 for the plasmid 2 (pAPEC_078.2), and CP010318 for the plasmid 3 (pAPEC_078.3). Download Table S3, XLSX file, 0.1 MB. Copyright © 2022 Yair et al. 2022 Yair et al. https://creativecommons.org/licenses/by/4.0/ This content is distributed under the terms of the Creative Commons Attribution 4.0 International license.

Because RBPs generally interact with their target molecules via RNA motifs or structures (2), we searched for a preferential binding site for CspC and CspE. A CMFinder (19) search for common secondary structures gave no significant results. When using the MEME Suite (20), we detected a putative CUG RNA motif, found in 232 CspC peaks (Fig. 2A). For CspE, an extended CUGXA motif was found in 24 peaks, and a CAG motif was found in 84 peaks (Fig. 2B). For peaks shared between the two CSPs, we detected a GXUCUG motif, found in 38 peaks (Fig. 2C). Taken together, the CLIP analysis revealed common peaks for CspC and CspE with preferential occurrence in respiratory chain and oxidative stress genes. In addition, a RNA binding motif, shared by both proteins was detected indicating a potential sequence recognition for CspC and CspE when they bind their targets.

### Functional analysis of potential CspC and CspE targets.

**FIG 3:**
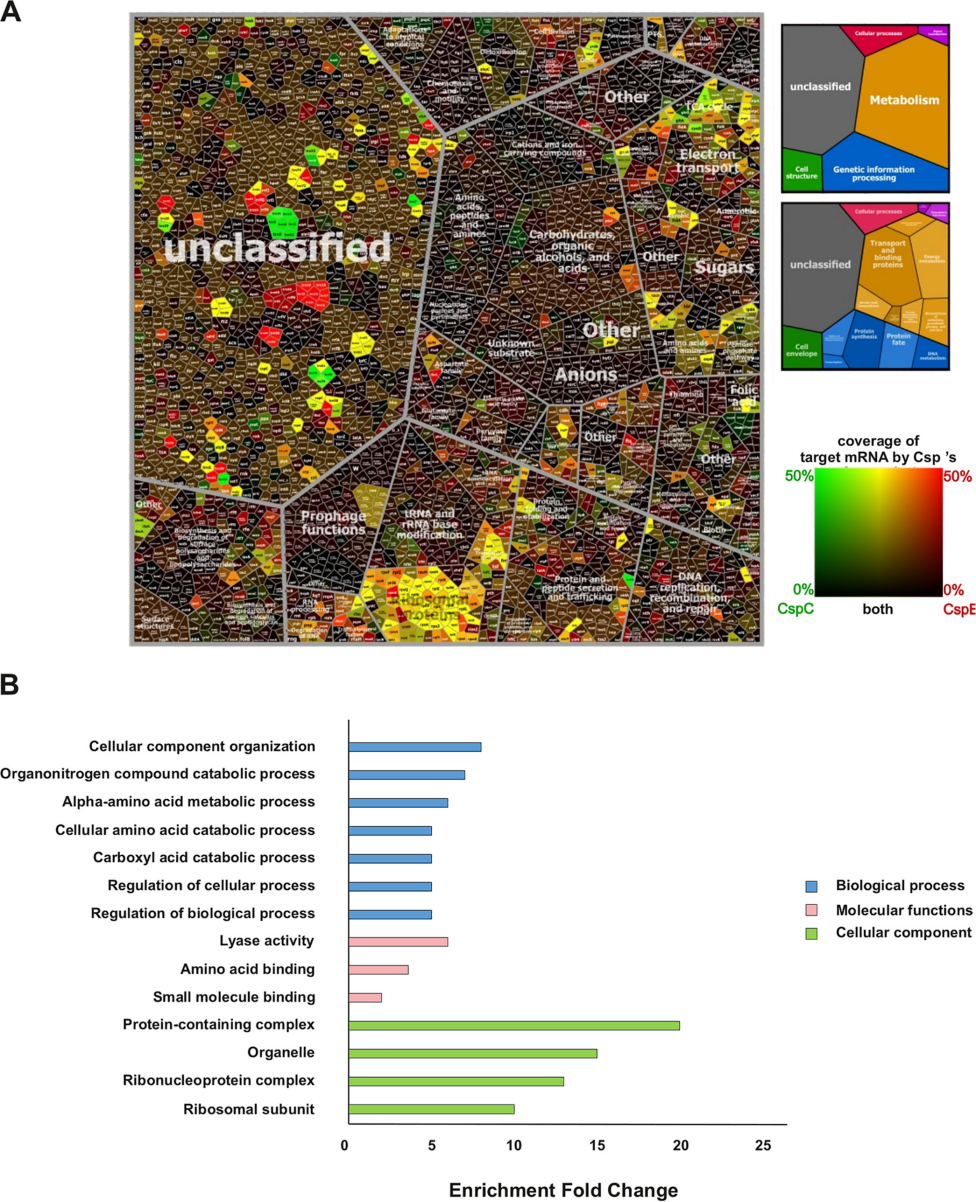
(A) Voronoi Tree map of transcripts bound by CspC and CspE in ExPEC O78-9 based on CLIP-seq analysis. Each cell represents one quantified transcript found bound to CspC, CspE, or both, and transcripts encoding functionally related proteins are subsumed in convex-shaped parental categories of increasing levels based on TIGR gene classification. The ratios of binding data were color coded by using a divergent color gradient. (B) Gene ontology enrichment analysis performed by Panther where three main annotation sets are represented (biological process, molecular function, cellular component). The *x* axis represents the enrichment fold (*P*-value <0.05) of each pathway. The *y* axis represents the enriched pathways, classified by color based on their annotation set.

To obtain an overview of transcript binding by CspC and CspE and better understand their biological functions, we compiled Voronoi tree maps from the CLIP-seq data. In the Voronoi tree maps, transcripts are clustered according to their functional classification as obtained from the TIGRFAM classification (21), and functionally related targets are clustered. The percent of coverage of target mRNA by CspC/CspE were color-coded based on a divergent color gradient (from green for maximal coverage of transcript by CspC to red for maximal coverage of transcript by CspE, and black for minimal coverage). This analysis indicated that transcripts associated with CspC and CspE were enriched mainly for functions in protein synthesis, transcription, and energy metabolism (Fig. 3A). A large cluster of ribosomal genes was found to be bound by either CspC, CspE, or both, which aligns with the peak enrichment found in the correspondent genomic location (Figs. S1C and S2C). A closer look at the shared targets of the CSPs was performed using Panther (22) gene ontology (GO) enrichment analysis with the three main annotation hierarchies: biological process, molecular function, and cellular component (Fig. 3B). Generally, transcripts associated with the CSPs were found to be enriched for biological processes such as cellular component organization, regulation of cellular, and biological, metabolic, and catabolic processes which agree with the Voronoi analysis. In terms of molecular functions, enrichment was found in lyase activity and amino acid and small molecule binding. Lastly, enriched cellular components included protein containing complexes, organelles, ribonucleoprotein complexes, and ribosomal subunits.

### Identification of potential virulence-related transcripts bound by CspC and CspE.

The binding analysis also pointed to several potential targets involved in pathogenesis. A full list is presented in Table 1 and includes transcripts of targets like *fur* and *ryhB*, involved in iron metabolism, which are known to be important for virulence. The list also contains several genes (*iutA*, *hlyF*, and *traT*) encoded on the ColV plasmid, of which TraT is known to mediate complement resistance (23).

**TABLE 1:** Virulence and iron metabolism-associated genes found in CLIP-seq

	Gene	Product	Location	Bound by	*P* value (adjusted)
Iron metabolism	*fepD*	ferric enterobactin transport system permease	Genomic	CspC	0.03
	*fur*	ferric uptake regulation protein Fur	Genomic	CspC	0.0004
	*ryhb*	small noncoding RNA RyhB	Genomic	Both	0.05
Virulence	*ompA*	outer membrane protein A	Genomic	CspC	0.041
	*crl*	transcriptional regulator of cryptic *csgA* gene for curli surface fibers	Genomic	CspC	0.049
	*tdcA*	transcriptional activator of *tdc* operon	Genomic	Both	0.0006 for CspC, 0.056 for CspE
	*clpX*	ATP-dependent specificity component of ClpP serine protease	Genomic	Both	0.001 for CspC, 0.02 for CspE
Iron metabolism	*iutA*	ferric aerobactin receptor precursor IutA	pColV	CspC	0.015
Known virulence	*hlyF*	hemolysin F protein HlyF	pColV	CspC	0.047
	*traT*	TraT complement resistance protein precursor	pColV	CspC	0.001

The list of potential targets involved in pathogenesis also includes genes with some evidence for involvement in virulence, such as *tdcA* and *clpX*. TdcA is the activator of the *tdcABCDEFG* operon, which is involved in the transport and metabolism of l-threonine and l-serine (24). All members of the operon were found to be bound by both CSPs. This includes the aforementioned *tdcA*, a member of the LysR family of transcription factors (25). Two genes of the *tdc* operon, *tdcA* and *tdcB*, were previously hypothesized to contain a binding motif for CspC (3), but no *in vivo* evidence has been shown so far. The *clpX* messenger was found to be bound by both CSPs (padj < 0.05). ClpX, together with ClpP, forms an ATP-dependent protease, responsible for elimination of misfolded proteins and interacting regulatory proteins (26). ClpXP are positive regulators of locus of enterocyte effacement (LEE) in Enterohemorrhagic E. coli (EHEC) O157 (27).

### Effect of CspC and CspE on target transcript levels.

**FIG 4:**
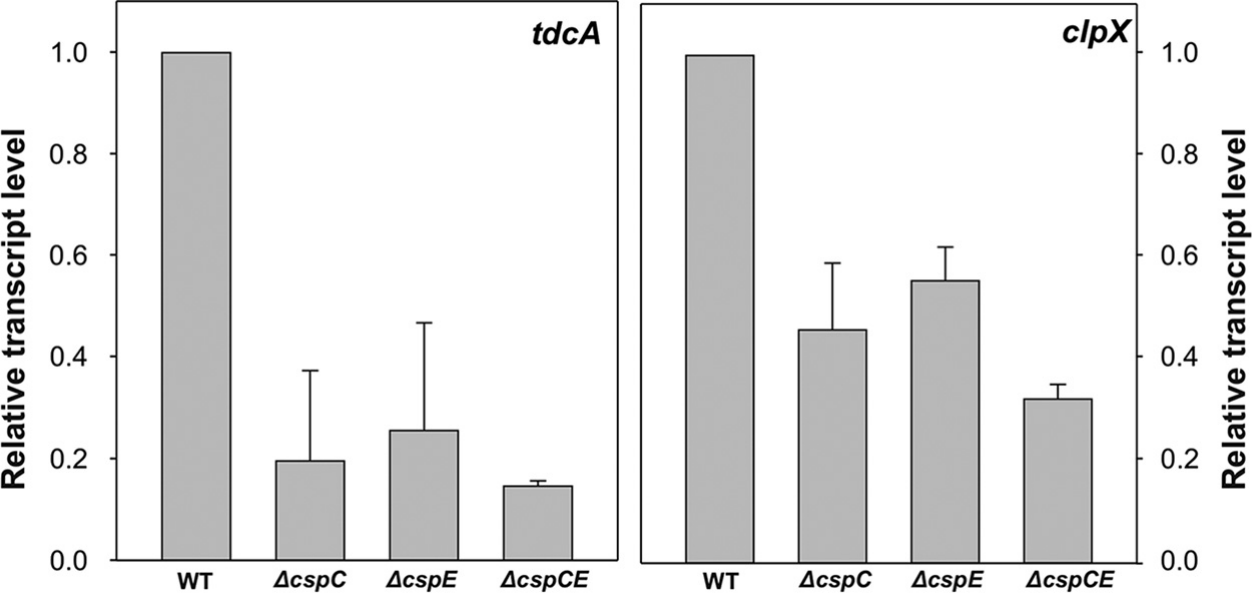
Levels of *clpX* and *tdcA* mRNA in single and double deletion mutants of *cspC* and *cspE* (A and B, accordingly). Cultures were grown at 37°C to OD _600 nm_ =1.4 and samples were taken for RNA extraction. Expression of target mRNA was determined using RT-PCR, and is normalized to the expression in the wild-type strain. The 16 S gene was used as a housekeeping gene for normalization. The data represents average results of three independent experiments.

As CspC and CspE are RNA chaperones, it is expected that their binding affects the stability of their target transcripts. To study this, we constructed single and double deletions of *cspC* and *cspE* (Δ*cspC*, Δ*cspE* and Δ*cspC*Δ*cspE*, accordingly) in O78-9 and examined the transcript levels of *tdcA* and *clpX.* The results are presented in Fig. 4 and indicate that the transcript levels of the two genes were lower in the mutants, compared with the wild type. The level of *clpX* transcript was reduced by 50% for the single mutants and 70% for the double mutant. For *tdcA*, around 80% reduction was observed for the single and double mutants (Fig. 4).

In order to further investigate the nature of CspC and CspE regulation on these targets, a more precise study of transcript stability was performed by determining the residual level of the selected transcript following addition of the transcription inhibitor Rifampicin in the Δ*cspC*Δ*cspE* mutant compared with the wild type after 4 and 6 min. A deletion of both proteins resulted in 32-fold decreased stability for *tdcA* and around 10-fold for *clpX* (Table 2).

**TABLE 2:** Effect of deletions in *cspC* and *cspE* on transcripts of *clpX* and *tdcA*^*a*^

Strain	Wild type			*ΔcspCE*		
Minutes after Rifampicin treatment	0	4	6	0	4	6
*tdcA* transcript level	1	1.04	0.98	1	0.11	0.03
*clpX* transcript level	1	0.4	0.14	1	0.13	0.01

a Cultures were grown at 37°C to OD600 = 1.4. Rifampicin was added (500 μg per ml) and samples were taken for RNA extraction at 0, 4, and 6 min. mRNA levels of *clpX* and *tdcA* were determined by RT-PCR and normalized to the expression in the wild type. The 16S gene was used as a normalizing gene. The data represents average results of two independent experiments in duplicates.

### The *tdcA* and *clpX* genes are important for virulence.

**FIG 5:**
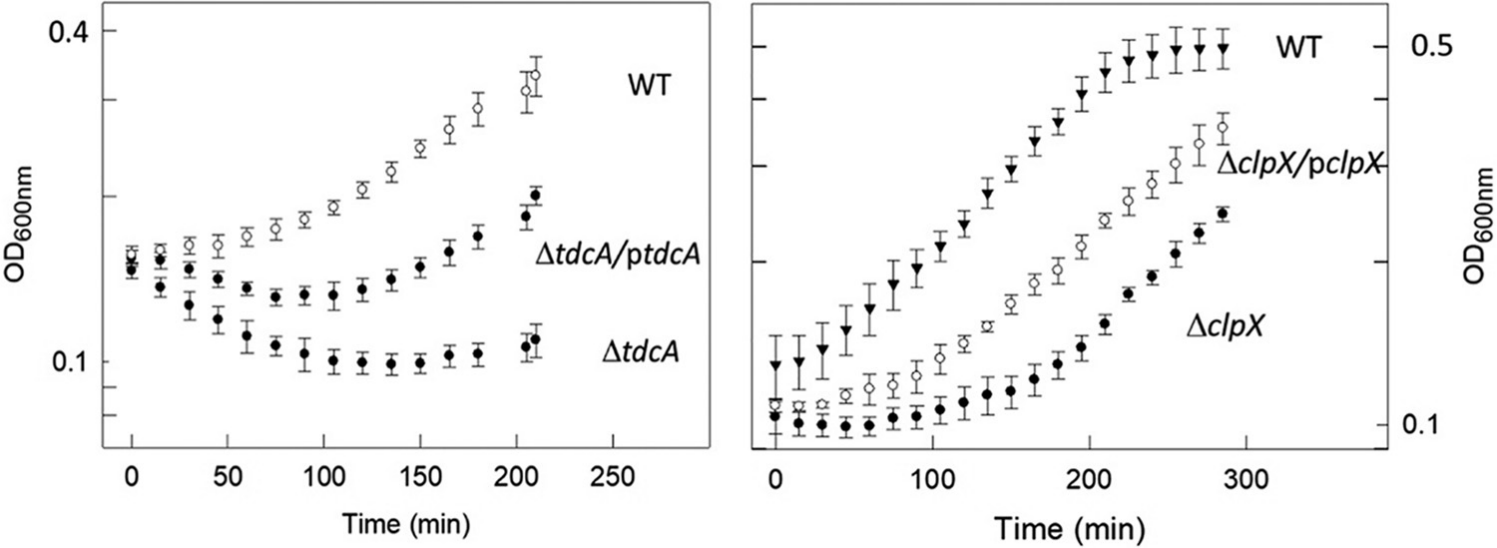
The *clpX* and *tdcA* genes are essential for serum survival. Cultures of O78-9 (wild type) and its deletion mutants Δ*clpX* or Δ*tdcA* were grown in Davis minimal medium to OD_600 nm_ = 0.4 and exposed to 40% serum (human, Sigma). The effect of the serum was determined by measuring growth as turbidity at OD_600 nm_.

In order to determine whether *tdcA* and *clpX* are important for virulence, we constructed deletion mutants of these genes and determined the effect of the deletion on serum sensitivity. The ability to resist serum is required for causing sepsis and for virulence (28, 29). Cultures were exposed to 40% human serum and the effect of serum on growth was monitored by measuring turbidity. The results presented in Fig. 5 indicate that the deletion mutants were serum sensitive and the sensitivity could be partially complemented by the presence of the respective genes on a plasmid.

### CspC and CspE are involved in serum resistance.

**FIG 6:**
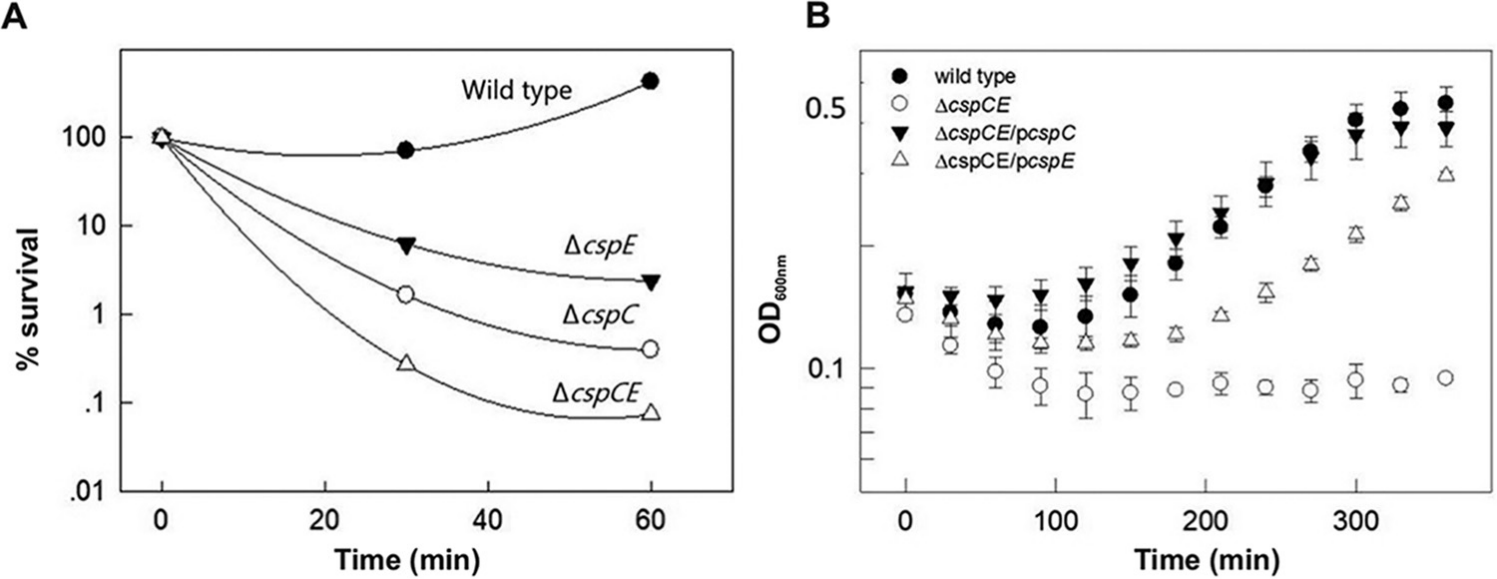
CspC and CspE are involved in serum resistance. The experiment was performed as described in Fig. 5. The effect of the serum was determined by viable count (A) or by measuring growth as turbidity at OD _600 nm_ (B). For viable count the bacteria were diluted in LB and 10 μL droplets were plated on LB agar plates. The plates were incubated overnight at 37°C and survival rate was calculated in comparison the wild type. The bacteria used –O78-9 (wild type), its double deletion mutant Δ*cspC cspE*, and the double deletion mutants complemented by *cspC* or *cspE* on pBAD plasmid (55).

The involvement of CspC and CspE in the regulation of transcripts of virulence-related genes suggested that these proteins are important for virulence in ExPEC. To determine the role of CspC/E in resistance to serum, cultures of the wild type as well as the single and double deletion mutants were exposed to 40% serum. The effect of serum was determined by viable counts (Fig. 6A) and by growth, as measured by turbidity (Fig. 6B). The results indicated that deletion of either *cspC* or *cspE* resulted in a significant loss of viability in the presence of serum, compared with the wild type. The double deletion mutant had the lowest survival rate upon exposure to serum, indicating a cumulative effect of the double deletion (Fig. 6A). The serum sensitivity could be complemented by the addition of the relevant genes on a plasmid. Interestingly, the double deletion mutant could be fully complemented by *cspC*, while with *cspE* complementation was partial (Fig. 6B).

## DISCUSSION

In this study, we aimed to better understand the global role of the RNA chaperones CspC and CspE in modulating transcript stability in E. coli, with emphasis on their role in pathogenesis. We used CLIP-seq to identify the transcripts bound to these two chaperones. Our analysis indicated that both CspC and CspE are involved in binding of a large variety of targets. In a similar analysis that we performed in E. coli K-12 MG1655, we identified a total of 713 potential CspC-binding targets; 321 were shared by the two E. coli strains (unpublished) and 158 of which are also present in Salmonella (15). Our results also include some of the genes, i.e., *katG*, *tnaA*,*treC*, *uspA*, *rpoS*, and *malE*, previously identified as CspC targets (5).

Functional enrichment analysis of transcripts bound by CspC and CspE indicates that they are involved in various pathways, particularly in protein synthesis, transcription, and energy metabolism (Fig. 3). These results point toward several interesting targets to be further investigated, such as the interactions of the CSPs with the *suc*, *atp*, and *cyd* operons. These operons encode key players in energy metabolism and in the aerobic respiratory chain, which is important for virulence of ExPEC (30–32). In Salmonella, CspC and CspE were shown to bind transcripts functionally enriched in pathways involved in RNA polymerase, transport, ribosome, sugar biosynthesis, motility, and more. They were also found to bind members of the *suc* and *atp* operons, but not the *cyd* operon (15).

Another interesting finding is the interactions of the CSPs with several previously unidentified intergenic regions of O78-9 (Tables S1 and S2). Both CSPs were found to bind sRNAs (30 for CspC, 11 for CspE) and interact with intergenic regions (166 for CspC, 64 for CspE) (Fig. S1A; Fig. S2A). Because CspC and CspE were previously predicted to bind sRNAs as well as mRNAs (15), this could help to identify additional sRNAs of E. coli, as well as novel regulatory modes of action.

Many of the targets are bound by both CspC and CspE, supporting previous findings on the redundant nature of CspC and CspE (6). It should be noted that in many of these cases a deletion of one of these chaperones can be compensated to some extent by the presence of the other, and only a deletion of both results in a severe phenotype (Fig. 6). Redundancy is also observed for the RNA motif found common in peaks of CspC and CspE. The relatively small motif size seems to indicate that, as previously reported, CSPs may bind many ligands with low affinity (5). This motif, an extended CUG sequence, has been reported to be the RNA binding motif of the CUGBP1 protein, known to regulate pre-mRNA alternative splicing and involved in mRNA editing and translation in humans (33). Intriguingly, CSPs in bacteria are composed of two cold shock domains, RNP1 and RNP2, domains also found within the three RRM domains of the CUGBP1 protein in humans (34, 35).

The binding of CspC and CspE can protect the target transcripts from degradation (15). We could show that the cellular level of two targets—*clpX* and *tdcA*—are considerably reduced in strains lacking CspC and CspE and so is their transcript stability (Fig. 2 and 3). This result supports the notion that the CSPs protect target transcripts against cleavage by cellular RNases, such as RNase E, as shown previously in Salmonella (15).

The results obtained with the *tdcA* transcript are especially striking, as this transcript is very stable. The regulation of the *tdc* operon is complex, and involves CRP, IHF, TdcR, and other elements (36–38). The transcription of the *tdcABCDEFG* operon is known to be relatively rifampicin-insensitive (39), indicating high transcript stability. However, our experiments suggest that this stability is due to the binding of the RNA chaperones, as the absence of CspC and CspE resulted in 32-fold decrease in *tdcA* transcript stability. Our results suggest an additional mechanism of regulation for the *tdc* operon, which involves CspC and CspE.

TdcA plays a role in the virulence of *S. Typhimurium*, in which a *tdcA* mutation affects invasion of epithelial cells by affecting the expression of motility genes, such as *fliC* and *fliZ* (40). In E. coli EHEC O157:H7, *tdcA* is a possible regulator of OmpA, and deletion of *tdcA* leads to hyper-adherence to various cell types (41, 42). Evidence from Klebsiella pneumonia also suggest that the *tdc* locus is involved in virulence and infection (43). We could show the direct involvement of TdcA in virulence, as its deletion results in serum sensitivity of E. coli O78-9 (Fig. 5).

The strong impact that deletion of either CspC or CspE has in reducing serum resistance is highly intriguing. Surviving serum is a prerequisite for causing sepsis. Therefore, the fact that septicemic bacteria require CspC and CspE for serum resistance indicates that these factors are essential for virulence. These results are compatible with results indicating that that a *ΔcspCE* mutant of Salmonella was avirulent in a mouse model (15). The serum sensitivity resulting from deletions of the *cspC* and *cspE* genes is probably due to the loss of protection of transcripts for proteins that are essential for serum survival. One class of such transcripts includes *fur* and *ryhB*, which we identified as CSPs targets. Fur is the master regulator of cellular iron homeostasis and RyhB is a small noncoding RNA that downregulates the expression of genes encoding iron‐containing proteins, and is regulated by Fur (44) while Fur itself is also regulated by RyhB (45). RyhB is known to be bound by the RBP Hfq (46), which in turn interacts with CspC (47) (Fig. 1B). Our results point toward involvement of the CSPs in iron metabolism, possibly via interactions with Fur and RyhB. There are also additional transcripts for proteins that are essential for serum survival includes proteins but are not involved in iron homeostasis. Two such proteins are ClpX and TdcA, which are essential for serum survival (Fig. 5).

The requirement for CspC and CspE for stabilization of transcripts of virulence-related genes suggested that these two RNA chaperones are essential for pathogenesis of ExPEC. Indeed, deletion of the *cspC* or *cspE* genes rendered the bacteria completely serum sensitive. This result further emphasizes the central role of these two chaperons in stress adaptation and endurance of extreme conditions. Here, we also show the synergy between these two chaperones, as a deletion of one of them can be partially compensated by the presence of the other in stabilizing some of the transcripts (Fig. 5). These results are compatible with previous findings (6, 12) showing overlapping functions of CspC and CspE in regulating some—but not all—transcripts. Interestingly, the serum sensitivity of the double deletion mutant could be fully complemented by the addition of the *cspC* gene, but only partially complemented by the *cspE* gene (Fig. 6B). The unique contribution of CspC in protecting against serum exposure is consistent with previous results showing the essentiality of this chaperone in protecting against heat shock damage (6, 12). These finding provide further support for the hypothesis that CspC levels regulate transcription upon exposure to environmental stress while CspE acts more as a “housekeeping RNA chaperone” under general stress conditions.

The results presented here provide further indication for the global importance of CspC and CspE for E. coli physiology, stressing the importance of these two proteins for virulence of ExPEC. The role of CspC and CspE in ExPEC pathogenesis marks them as potential targets for novel antimicrobials agents.

## MATERIALS AND METHODS

### Strains, growth conditions, and media.

The E. coli strains used in this study are listed in Table 3. Bacteria were routinely grown at 37°C with aeration in Luria-Bertani (LB) broth or Davis minimal medium (48). For viable count, samples were diluted and plated on LB agar plates for colony counts.

**TABLE 3:** Strains used in this study

Strain	Description	Reference
O78-9	Isolate of E. coli O78	29
O78-9 CspC FLAGx1	O78-9 with a chromosomally flag-tagged *cspC* Km^R^	This study
O78-9 CspE FLAGx1	78-9 with a chromosomally flag-tagged *cspE* Km^R^	This study
O78-9Δ*clpX*	O78-9 deleted for *clpX*. Km^R^	This study
O78-9Δ*clpX*/p*clpX*	O78-9 deleted for *clpX*. Km^R^ carrying pBAD plasmid (56) with *clpX* gene	This study
O78-9Δ*tdcA*	O78-9 deleted for *tdcA*. Km^R^	This study
O78-9Δ*tdcA*/p*tdcA*	O78-9 deleted for *tdcA*. Km^R^ carrying pBAD plasmid (56) with *tdcA* gene	This study
O78-9Δ*cspC*	O78-9 deleted for *cspC*. Km^R^	This study
O78-9Δ*cspE*	O78-9 deleted for *cspE*. Km^R^	This study
O78-9Δ*cspC* Δ*cspE*	O78-9 deleted for *cspC* and *cspE*. Km^R^	This study
O78-9Δ*cspC* Δ*cspE*/p*cspC*	O78-9 deleted for *cspC* and *cspE*. Km^R^ carrying pBAD plasmid (56) with *cspC* gene	This study
O78-9Δ*cspC* Δ*cspE*/p*cspE*	O78-9 deleted for *cspC* and *cspE*. Km^R^ carrying pBAD plasmid (56) with *cspE* gene	This study

### Construction of deletion mutants.

Deletions of *cspC*, *cspE*, *tdcA*, and *clpX* were constructed as previously described (49). Briefly, electro-competent cells were transformed with plasmid pKD46. The transformants were grown in LB medium with ampicillin, induced with arabinose, and made competent for electroporation. A linear PCR product was constructed on pKD4 template of a kanamycin resistance cassette flanked by FLP recognition target (FRT) sequences from the designated deletion region (oligonucleotide primers are listed in Table S4). Kanamycin-resistant cells were picked and examined by colony PCR. The pKD46 plasmid was removed by growth on LB plates at 42°C overnight. The final deletion was verified by sequencing.

10.1128/msystems.00086-22.6 TABLE S4 Primers used in this study. Download Table S4, DOCX file, 0.01 MB. Copyright © 2022 Yair et al. 2022 Yair et al. https://creativecommons.org/licenses/by/4.0/ This content is distributed under the terms of the Creative Commons Attribution 4.0 International license.

### UV cross-linking, immunoprecipitation, and RNA purification.

CLIP-seq was performed as described before (17). Briefly, the cells were lysed and immunoprecipitated using specific antibodies and the bound RNA was trimmed by RNase digestion, leaving only the region surrounding the direct interaction site. The RNA was then radiolabeled, the labeled complexes separated on SDS-PAGE (Fig. 1B), and transferred to a nitrocellulose membrane. The RNA was released by Proteinase K and subjected to high-throughput sequencing. For each biological replicate, 200 mL bacterial culture were grown to OD_600_ of 2.0. Half of the culture was treated directly and half was irradiated with UV‐C light at 800 mJ/cm^2^.

### cDNA library preparation and sequencing.

cDNA libraries were prepared as described elsewhere (17) using the NEBNext Multiplex Small RNA Library Prep Set for Illumina (#E7300, New England Biolabs) according to the manufacturer’s instructions. High-throughput sequencing was performed at the Core Unit Systems Medicine, University of Würzburg. The 24 cDNA libraries were pooled on an Illumina NextSeq platform, and sequencing done for single-end 1 × 75 bp reads. Sequencing yielded over 10 M reads per sample.

### Processing of sequence reads, mapping, and peak calling.

Peak calling was performed using the adaptive approach implemented in the tool PEAKachu (https://github.com/tbischler/PEAKachu), similarly to as described elsewhere (18). Reads were trimmed for adaptors and a minimum read quality of 20 using cutadapt 1.15 (50). Reads were mapped to O78-9 genome and plasmids p789-1, p789-2, and p789-3 (CP010315.1, CP010316 [p789-1], CP010317, and CP010318, respectively) obtained from NCBI using READemption version 0.3.7 (51). Only uniquely mapping reads were retained, and used as input for PEAKachu in adaptive peak calling mode using the default DESeq normalization between conditions. All data has been deposited on GEO with accession number GSE205384.

### Targets clustering, enrichment analysis, and Voroni tree map visualization.

The HMMER (52) algorithm was used for predicting gene functions according the TIGRFAM classification, as previously described (53). Visualization was done with Voronoi tree maps (54), in which all functionally assigned genes are shown in a hierarchically organized and space-filling manner (Fig. 3). The polygons on the last level represent all of the functionally assigned genes, and related genes are pinpointed in close proximity. CLIP-seq transcript data were mapped by using a divergent color gradient starting with black for nonbound targets, green for targets bound only CspC, and red for targets bound only by CspE.

### RNA extraction.

Cultures were grown as described above until they reached an OD_600_ of 1.2 to 1.4. Then, 500 μL were mixed with 1 mL of RNAprotect bacterial reagent (Qiagen) at room temperature. RNA purifications were conducted using the RNeasy minikit (Qiagen) according to the manufacturer’s instructions. DNase treatment was performed using RNase-free DNase (Qiagen) according to the manufacturer’s instructions.

### Real-time PCR.

For real‐time PCR (RT-PCR), 1 μg of total RNA was reverse‐transcribed using random hexamers (Promega) by the ImProm II reverse transcriptase (Promega). Each RT-PCR contained 500 ng of cDNA, 100 nM each gene‐specific primer, and 1x qPCRBIO Fast qPCR SyGreen Blue Hi-ROX mix (PCRBIOSYSTEMS), and DDW added to a final volume of 20 μL. Each primer specific reaction was done in triplicates. Reactions were run on a CFX Connect Real-Time PCR Detection System (BIO-RAD) using the standard cycling parameters. Relative expression was calculated with the ΔΔCq calculation (55). Oligonucleotide primers used in this study are listed in Table S4.

### Transcript stability assays.

Bacterial cultures were grown at 37°C to OD_600_ =1.2 to 1.4. Transcription was arrested with rifampicin (500 μg/mL final concentration) and samples were taken at time zero and additional time points. RNA was extracted, reverse-transcribed and transcript levels were determined using RT-PCR.

### Serum survival assay.

Overnight cultures were grown in Davis minimal medium and diluted in the morning to OD_600_ = 0.05. Cultures were grown for about 3 h, until logarithmic stage (OD_600_ = 0.4) and diluted to OD_600_ = 0.1, then exposed to 40% human serum (Sigma). At *t* = 0 and after 30, 60, and 120 min, 100 μL aliquots of each culture were collected to determine the number of viable cells by serial dilutions and plating onto LB agar. The data represents average results of three independent experiments.

### Growth in the presence of serum.

Overnight culture grown in Davis minimal medium were diluted 1:20 into a sterile 96-well plate. Human serum (Sigma) was added to concentrations of 0% or 40%. Growth was determined using a BioTek Eon plate reader, and turbidity at OD = 600 nm was measured every 15 min for at least 2 h.

### Complementation of deletion mutants.

Recombinant plasmids carrying the *clpX*, *tdcA*, *cspC*, or *cspE* gene were constructed by cloning PCR-amplified DNA fragments (primers listed in Table S4) into pBAD24 vector (56) using the HiFi DNA assembly cloning kit (NEBuilder).
