# Supplementary material for: Cellular RNA Targets of Cold Shock Proteins CspC and CspE and Their Importance for Serum Resistance in Septicemic Escherichia coli
Source: mSystems. 2022 Jun 13;7(4):e00086-22. doi: 10.1128/msystems.00086-22 (PMC9426608; doi:10.1128/msystems.00086-22)
Supplement: FIG S1 [file msystems.00086-22-sf001.pdf]

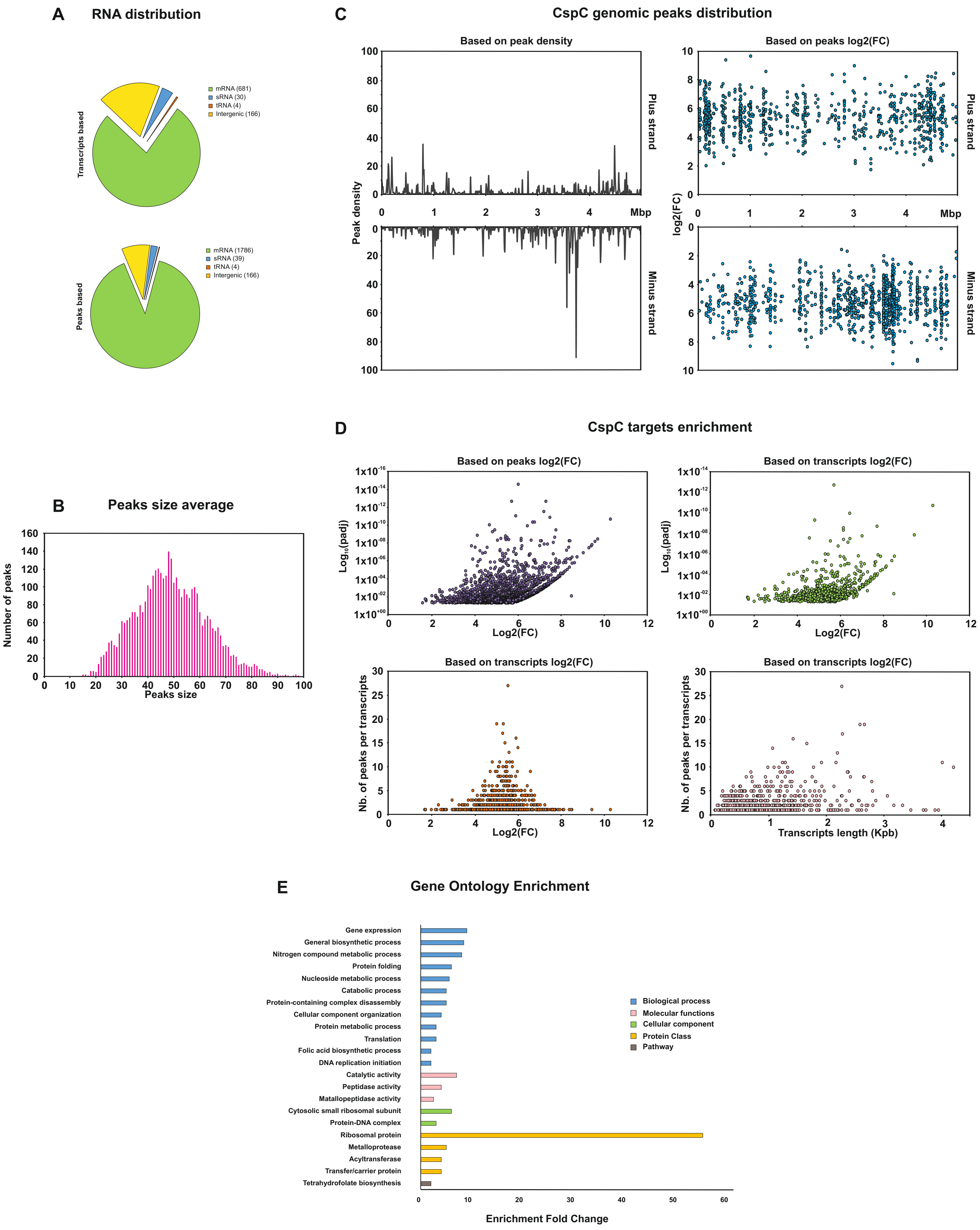

Supplementary figure 1.
