# Supplementary figures and images for: Cellular RNA Targets of Cold Shock Proteins CspC and CspE and Their Importance for Serum Resistance in Septicemic Escherichia coli
Source: mSystems. 2022 Jun 13;7(4):e00086-22. doi: 10.1128/msystems.00086-22 (PMC9426608; doi:10.1128/msystems.00086-22)

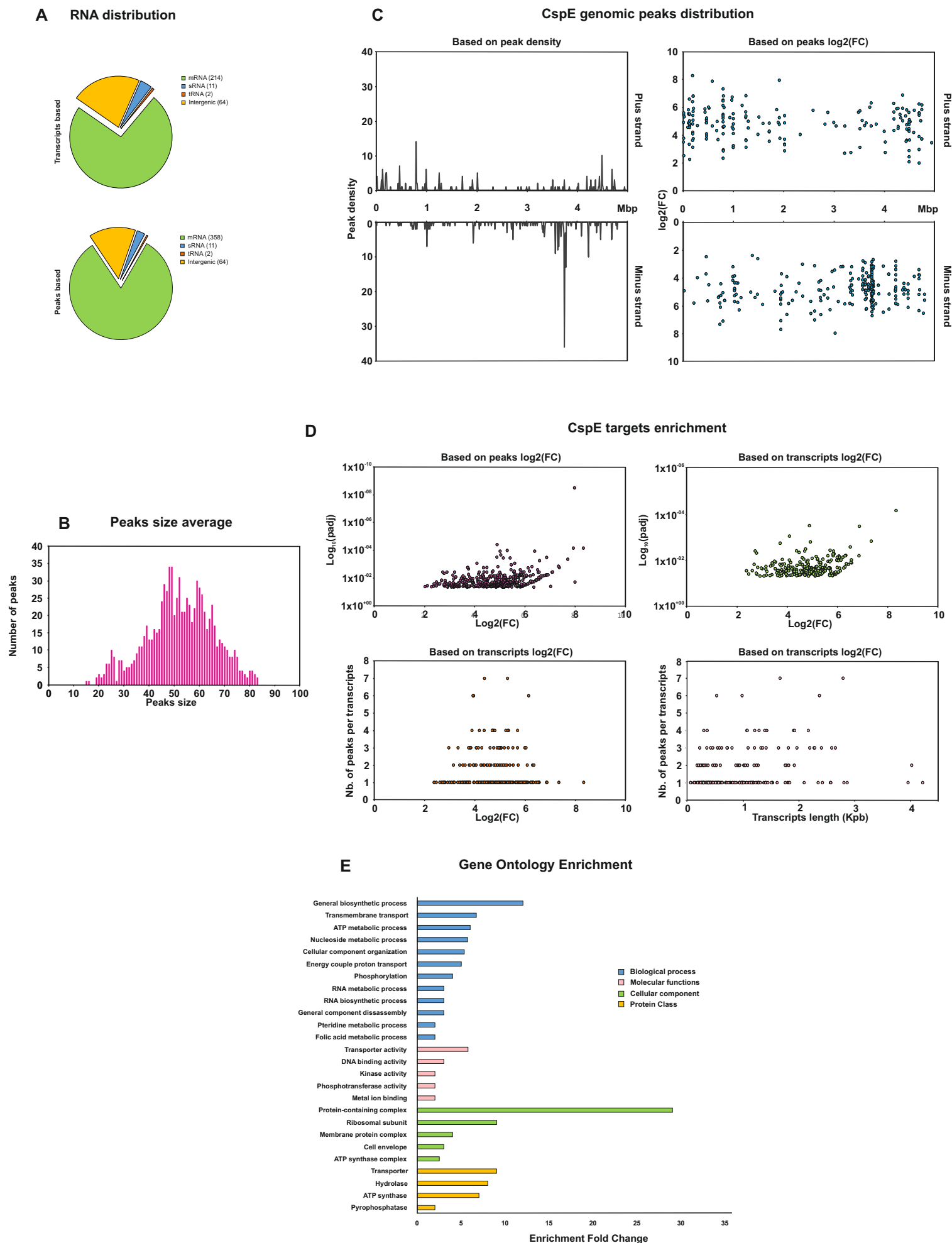

**Supplementary figure 2.**

Supplement: FIG S2 [file msystems.00086-22-sf002.pdf]
