# Supplementary material for: Cellular RNA Targets of Cold Shock Proteins CspC and CspE and Their Importance for Serum Resistance in Septicemic Escherichia coli
Source: mSystems. 2022 Jun 13;7(4):e00086-22. doi: 10.1128/msystems.00086-22 (PMC9426608; doi:10.1128/msystems.00086-22)
Supplement: TABLE S4 [file msystems.00086-22-st004.docx]

**Table 4s**. Primers used in this study

| Name | Sequence |
| --- | --- |
| P1*cspC* | CGGGCGAAGAAAACAATCTAAGGAATTTTTCAAATGGTGTAGGCTGGAGCTGCTTC |
| P2*cspC* | AAGCGTATTCACACTTCAGATCAGTGGATTCGATCACATATGAATATCCTCCTTAG |
| P1*cspE* | TTTGTGTCTATTTTTCATGTAAAGGTAATTTTGATGGTGTAGGCTGGAGCTGCTTC |
| P2*cspE* | ATTAAGCGGGTTTTGAATTCTTGCTGACGTATCTTACATATGAATATCCTCCTTAG |
| P1*clpX* | ATGACAGATAAACGCAAAGATGGCTCAGGCAAATTGGTGTAGGCTGGAGCTGCTTC |
| P2*clpX* | TTCACCAGATGCCTGTTGCGCTTCCGGCTTGCCATACATATGAATATCCTCCTTAG |
| P1*tdcA* | TAGGTCGTTATGAGCACTATTCTTCTTCCGAAAACGGTGTAGGCTGGAGCTGCTTC |
| P2*tdcA* | CTAACCAACTTCTATTAATTGCCTTCGTCTACACCCCATATGAATATCCTCCTTAG |
| *csp*Cup-Flag | CAGAAAGGTCCGGCAGCTGTTAACGTAACAGCTATCGACTACAAAGATGACGACGA |
| *csp*Cdown-Flag | TTGAAGCGTATTCACACTTCAGATCAGTGGATTCGACATATGAATATCCTCCTTAG |
| *cspE*up-Flag | GCCAAAGGCCCTTCTGCTGCAAACGTAATCGCTCTGGACTACAAAGATGACGACGA |
| *cspE*down-Flag | CTGATTAAGCGGGTTTTGAATTCTTGCTGACGTATCCATATGAATATCCTCCTTAG |
| RT *cspC* F | GGCTTCATTACTCCGGCTGA |
| RT *cspC* R | CCTTTCTGGCCGTCCTGAA |
| RT 16s F | AGCTAACGCGTTAAGTCG |
| RT 16s R | TAAGGTTCTTCGCGTTGC |
| RT *clpX* F | GAAGTGCGCAAGCTGATT |
| RT *clpX* R | TAATCGTCCAGGTGGTTG |
| RT *tdcA* F | CCGTTCCGAATCCATTAC |
| RT *tdcA* R | CAATCAATGAAGGAAAACCACAATCAATGAAGGAAAACCA |
| pBADclpX F | GTTTTTTTGGGCTAGCAGGAGGAATTCACCATGACAGATAAACGCAAAGA |
| pBADclpX R | CTTCTCTCATCCGCCAAAACAGCCAAGCTTAATTGTATGGGAATGGTTAA |
| pBADtdcA F | GTTTTTTTGGGCTAGCAGGAGGAATTCACCATGAGCACTATTCTTCTTCC |
| pBADtdcA R | CTTCTCTCATCCGCCAAAACAGCCAAGCTTGACAGACAGGTGGATTATTT |
| pBADcspC F | GTTTTTTTGGGCTAGCAGGAGGAATTCACC ATGGCAAAGATTAAAGGTCA |
| pBADcspC R | CTTCTCTCATCCGCCAAAACAGCCAAGCTTAAGCGTATTCACACTTCAGA |
| pBADcspE F | GTTTTTTTGGGCTAGCAGGAGGAATTCACCATGTCTAAGATTAAAGGTAA |
| pBADcspE R | CTTCTCTCATCCGCCAAAACAGCCAAGCTTTAAGCGGGTTTTGAATTCTT |
